# Acute Stress Increases Depolarization-Evoked Glutamate Release in the Rat Prefrontal/Frontal Cortex: The Dampening Action of Antidepressants

**DOI:** 10.1371/journal.pone.0008566

**Published:** 2010-01-05

**Authors:** Laura Musazzi, Marco Milanese, Pasqualina Farisello, Simona Zappettini, Daniela Tardito, Valentina S. Barbiero, Tiziana Bonifacino, Alessandra Mallei, Pietro Baldelli, Giorgio Racagni, Maurizio Raiteri, Fabio Benfenati, Giambattista Bonanno, Maurizio Popoli

**Affiliations:** 1 Department of Pharmacological Sciences, Center of Neuropharmacology and Center of Excellence on Neurodegenerative Diseases, University of Milano, Milano, Italy; 2 Department of Experimental Medicine, Section of Pharmacology and Toxicology, University of Genova, Genova, Italy; 3 Department of Neuroscience and Brain Technologies, The Italian Institute of Technology, Genova, Italy; 4 Istituto di Ricovero e Cura a Carattere Scientifico San Giovanni di Dio - Fatebenefratelli, Brescia, Italy; 5 Center of Excellence for Biomedical Research and National Institute of Neuroscience, Genova, Italy; 6 Department of Experimental Medicine, Section of Physiology, University of Genova and National Institute of Neuroscience, Genova, Italy; University of Parma, Italy

## Abstract

**Background:**

Behavioral stress is recognized as a main risk factor for neuropsychiatric diseases. Converging evidence suggested that acute stress is associated with increase of excitatory transmission in certain forebrain areas. Aim of this work was to investigate the mechanism whereby acute stress increases glutamate release, and if therapeutic drugs prevent the effect of stress on glutamate release.

**Methodology/Findings:**

Rats were chronically treated with vehicle or drugs employed for therapy of mood/anxiety disorders (fluoxetine, desipramine, venlafaxine, agomelatine) and then subjected to unpredictable footshock stress. Acute stress induced marked increase in depolarization-evoked release of glutamate from synaptosomes of prefrontal/frontal cortex in superfusion, and the chronic drug treatments prevented the increase of glutamate release. Stress induced rapid increase in the circulating levels of corticosterone in all rats (both vehicle- and drug-treated), and glutamate release increase was blocked by previous administration of selective antagonist of glucocorticoid receptor (RU 486). On the molecular level, stress induced accumulation of presynaptic SNARE complexes in synaptic membranes (both in vehicle- and drug-treated rats). Patch-clamp recordings of pyramidal neurons in the prefrontal cortex revealed that stress increased glutamatergic transmission through both pre- and postsynaptic mechanisms, and that antidepressants may normalize it by reducing release probability.

**Conclusions/Significance:**

Acute footshock stress up-regulated depolarization-evoked release of glutamate from synaptosomes of prefrontal/frontal cortex. Stress-induced increase of glutamate release was dependent on stimulation of glucocorticoid receptor by corticosterone. Because all drugs employed did not block either elevation of corticosterone or accumulation of SNARE complexes, the dampening action of the drugs on glutamate release must be downstream of these processes. This novel effect of antidepressants on the response to stress, shown here for the first time, could be related to the therapeutic action of these drugs.

## Introduction

Behavioral stress is recognized as a main risk factor for many diseases, including cardiovascular, metabolic and neuropsychiatric diseases. Among the latter, stress interacts with variable genetic background of vulnerability in pathogenesis of mood/anxiety disorders [1]. Exposure of rodents to various stress protocols produces many behavioral, neurochemical, neuroendocrine, concomitants observed in humans [2]–[4]. While animals subjected to chronic stress paradigms are often used as models of psychiatric pathology, it is also important to study the brain response to acute stress, because this may be particularly helpful in dissecting the molecular and cellular mechanisms involved [5]. Acute stress induces rapid changes in the release of neurotransmitters, hormones and cytokines that are adaptive, but may become damaging if the stress response is inadequate or excessive. Inappropriate stress response acts as a trigger, which may produce a vulnerable phenotype in genetically predisposed individuals and increase the risk for mental ilness [2], [3]. There is to date a lack of data on the management of acute stress and on possible treatments that may alleviate the distressing acute symptoms and prevent damaging long-term consequences [6]. It should also be considered that the effects of chronic stress are not simply an extrapolation of the effects of acute stress, and complex adaptive phenomena must be taken into account in the long run.

A number of studies have suggested that acute stress is associated with increased excitatory amino acid transmission in areas of the forebrain. Restraint stress, tail pinch, forced swimming, footshock, and anxiogenic drugs have been shown to increase the efflux of glutamate as measured by microdialysis *in vivo* [7]–[9]. However, although it is assumed that behavioral stressors can deeply affect homeostasis of glutamate transmission, it is not yet clear:

1. What is the effect of acute stress on exocytotic release of glutamate in limbic/cortical areas. Thus far this has been assessed mainly or exclusively by *in vivo* microdialysis. However, it has been argued that extracellular glutamate and GABA, as measured by microdialysis, do not fulfill the classical criteria for exocytotic release (tetrodotoxin sensitivity, calcium-dependency). Accordingly, a large portion of amino acid neurotransmitters measured by this way may be of non-neuronal origin and possibly carrier-mediated or derived from glial metabolism [10]. Independent evidence (not from microdialysis) would be helpful to verify the outcome of acute stress on glutamate release and transmission.
2. What are the mechanisms whereby acute stress modifies glutamate release. Recent work has shown that stress rapidly increases the level of circulating stress-hormones and that in hippocampus (HPC) corticosterone (CORT) binds to membrane mineralocorticoid receptors (MR), which may rapidly induce the release of glutamate through non-transcriptional mechanisms [11], [12]. It is not known at present what changes in the mechanisms regulating glutamate release are affected by CORT.
3. What is the effect of psychotropics used for therapy of psychiatric disorders on the release of glutamate. A few studies have suggested that chronic antidepressant treatments reduce glutamate release in limbic/cortical areas in basal conditions [13]–[15]. In particular, we have found previously that chronic antidepressants reduce (K^+^)depolarization-evoked release of endogenous glutamate from synaptic terminals of HPC, with concomitant modifications in protein-protein interactions regulating assembly of the presynaptic SNARE (soluble *N*-ethylmaleimide-sensitive fusion protein attachment protein receptor) complex, that mediates fusion of synaptic vesicles, and reduction of complexes in presynaptic membranes [13], [16].

Here we found that acute footshock (FS)-stress up-regulates depolarization-evoked exocytotic glutamate release in prefrontal/frontal cortex (P/FC) but not HPC, by increasing the circulating levels of CORT, stimulating glucocorticoid receptors (GR) in P/FC, and inducing accumulation of SNARE complexes in presynaptic membranes. Moreover, analysis of excitatory postsynaptic currents (EPSCs) in pyramidal neurons of the prefrontal cortex (PFC), showed that stress induced dramatic changes in paired-pulse facilitation (PPF) and a slowdown of the EPSC kinetics, suggestive of both pre- and postsynaptic actions. Previous chronic administration of different antidepressants completely prevented the stress-induced up-regulation of glutamate release by a mechanism downstream of the elevation of CORT and SNARE complex accumulation, which involves a decrease in release probability.

## Results

### Depolarization-evoked release of glutamate from P/FC synaptosomes is increased after acute footshock stress

Immediately after the FS-stress session, the synaptosomes were purified from HPC and P/FC. Basal and 15 mM KCl-evoked release of endogenous glutamate and GABA were measured from synaptosomes in superfusion, the method of choice for the analysis of neurotransmitter release from a single family of nerve terminals, as described previously [13], [17]–[19]. We have previously shown that glutamate release measured by this way is essentially exocytotic, dependent on external calcium and independent on the function of membrane glutamate transporter [13].

**Figure 1:**
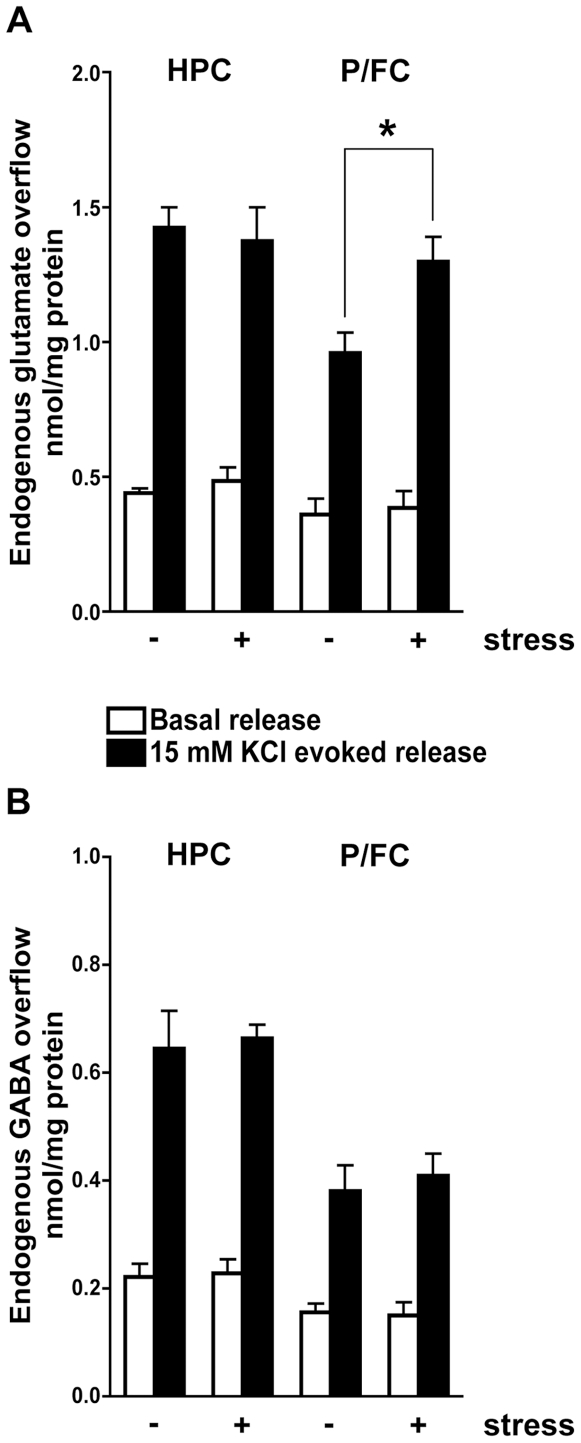
Basal and depolarization-evoked release of amino acid neurotransmitters from synaptosomes after acute footshock stress. A) Basal and 15 mM KCl evoked glutamate release from hippocampal (HPC) and prefrontal/frontal cortex (P/FC) synaptosomes of vehicle-treated (control, CNT) and subjected to acute footshock (FS)-stress (−/+ Stress) rats. Data are expressed as means±SEM. *p<0.05, two-tailed Student's t test). Open bars, basal release; filled bars, 15 mM KCl evoked release (n = 4–6 rats/group). B) Basal and 15 mM KCl evoked GABA release from HPC and P/FC synaptosomes of vehicle-treated (CNT) and subjected to FS-stress (−/+ Stress) rats. Data are expressed as means±SEM. Open bars, basal release; filled bars, 15 mM KCl evoked release.

Basal release of amino acid neurotransmitters from synaptosomes, as well as depolarization-evoked release of GABA, were unchanged in stressed rats (both areas) (Fig. 1A–B). Instead, acute stress exerted different effects on depolarization-evoked glutamate release in the two areas. While in HPC there were no changes, in P/FC depolarization-evoked overflow of glutamate was markedly and significantly increased in stressed rats (52%; p<0.05, Student's t test). This result was in line with previous findings of microdialysis studies [7]–[9], [20], and suggested that acute FS-stress selectively increases the release of glutamate (but not of GABA) in P/FC, and does not affect glutamate release in HPC.

### Chronic antidepressant treatments dampen the increase of glutamate release from P/FC synaptosomes induced by footshock stress

**Figure 2:**
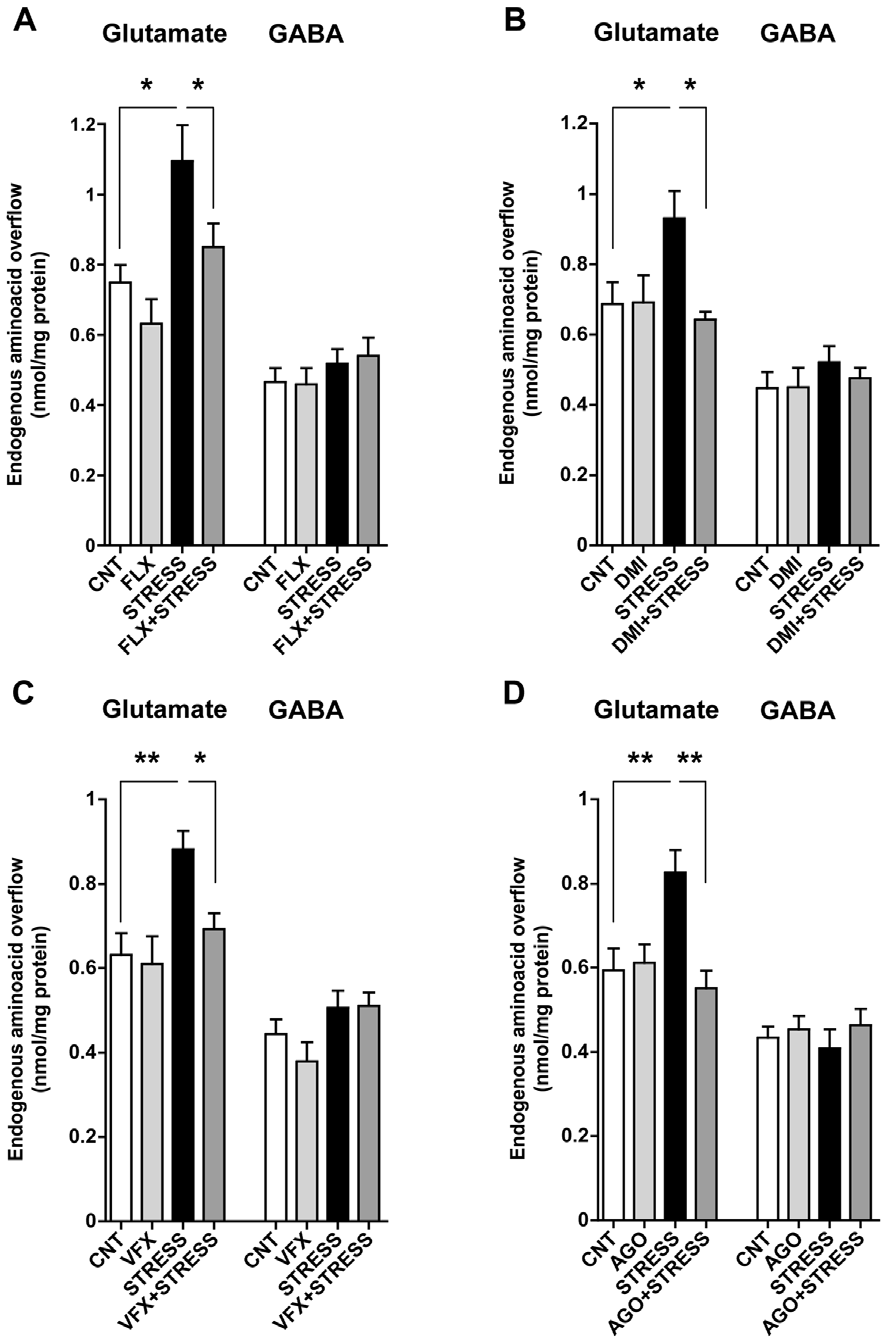
Effect of chronic antidepressant treatments on depolarization-evoked release of amino acid neurotransmitters from prefrontal/frontal cortex synaptosomes. A) 15 mM KCl evoked glutamate and GABA release from P/FC synaptosomes of vehicle-treated (CNT), chronically treated with fluoxetine (FLX), subjected to FS-stress (STRESS) or chronically treated with FLX and then subjected to FS-stress (FLX+STRESS) rats. Data are expressed as means±SEM. *p<0.05, Newman-Keuls post-hoc tests following one-way ANOVA (n = 6–9 rats/group). B) 15 mM KCl evoked glutamate and GABA release from P/FC synaptosomes of vehicle-treated (CNT), chronically treated with desipramine (DMI), subjected to FS-stress (STRESS) or chronically treated with DMI and then subjected to FS-stress (DMI+STRESS) rats. Data are as in A. *p<0.05, statistics as above. C) 15 mM KCl evoked glutamate and GABA release from P/FC synaptosomes of vehicle-treated (CNT), chronically treated with venlafaxine (VFX), subjected to FS-stress (STRESS) or chronically treated with VFX and then subjected to FS-stress (VFX+STRESS) rats. Data are as in A. *p<0.05; **p<0.01, statistics as above. D) 15 mM KCl evoked glutamate and GABA release from P/FC synaptosomes of vehicle-treated (CNT), chronically treated with agomelatine (AGO), subjected to FS-stress (STRESS) or chronically treated with AGO and then subjected to FS-stress (AGO+STRESS) rats. Data are as in A. **p<0.01, statistics as above.

We have shown previously that chronic treatments with different antidepressants reduce depolarization-evoked release of glutamate from HPC synaptosomes in basal conditions [13]. We asked here whether these drugs may also dampen the increase of glutamate release induced by acute stress in P/FC. We carried out two distinct cycles of treatments employing four different drugs (see Methods). In both studies, after 14 days of treatment with vehicle or drug, each rat group was randomly divided in two groups that were subjected to FS-stress, or sham-stressed (controls). As shown in Fig. 2A–D, contrary to our previous results in HPC [13], in P/FC none of the drugs significantly reduced depolarization-evoked glutamate release in the absence of stress. In both studies acute FS-stress induced a marked and significant increase in glutamate release (from 35–46%). Interestingly, the stress-induced increase in glutamate release was completely prevented in rat groups previously treated with each of the four drugs. One-way ANOVA showed significant effects in both study 1 (F_3,20_ = 6,419, p<0.01 in the fluoxetine [FLX] group [Fig. 2A] and F_3,27_ = 3.951, p<0.01 in the desipramine [DMI] group [Fig. 2B]) and study 2 (F_3,17_ = 6.726, p<0.01 in the venlafaxine [VFX] group [Fig. 2C] and F_3,31_ = 6.405, p<0.01 in the agomelatine [AGO] group [Fig. 2D]). No effect of either stress or drug treatments on GABA release was observed.

### The increase of glutamate release induced in P/FC by footshock stress is dependent on release of corticosterone and activation of glucocorticoid receptor

**Figure 3:**
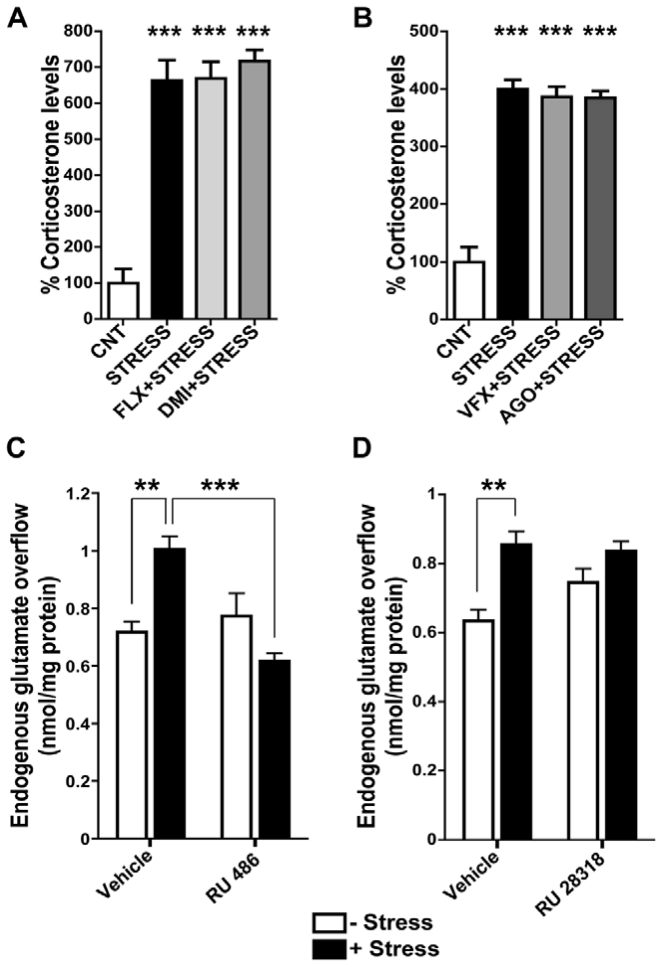
The increase of glutamate release induced in P/FC by footshock stress is dependent on release of corticosterone and activation of glucocorticoid receptor. A) Corticosterone serum levels in vehicle-treated (CNT), subjected to FS-stress (STRESS) and chronically treated with FLX or DMI and then subjected to FS-stress (FLX+STRESS; DMI+STRESS) rats. Data are expressed as means±SEM. *** p<0.001 vs CNT, Newman-Keuls post-hoc tests following one-way ANOVA (n = 5 rats/group). B) Corticosterone serum levels in vehicle-treated (CNT), subjected to FS-stress (STRESS) and chronically treated with VFX or AGO and then subjected to FS-stress (VFX+STRESS; AGO+STRESS) rats. Data are expressed as means±SEM. *** p<0.001 vs CNT, Newman-Keuls post-hoc tests following one-way ANOVA (n = 10 rats/group). C–D) Depolarization evoked glutamate release from P/FC synaptosomes of vehicle-treated (CNT) and subjected to FS-stress (STRESS) rats pretreated or not with RU 486 (C), a selective antagonist of glucocorticoid receptor or RU 28318 (D), a selective antagonist of mineralocorticoid receptor. Data are expressed as means±SEM. **p<0.01 and ***p<0.001, Bonferroni post-hoc tests following two-way ANOVA (n = 4 rats/group).

Compelling evidence has shown that rapid effects of stress on glutamate transmission and its plasticity in HPC are mediated by fast non-transcriptional action of CORT, seemingly due to the binding of CORT to membrane-located MR receptors [11], [12]. Here the levels of CORT were rapidly elevated in serum after FS-stress (Fig. 3A–B), whether rats were treated with antidepressants or not (F_3,15_ = 37,40; p<0.0001 in study 1 and F_3,33_ = 56,59; p<0.0001 in study 2) (for absolute CORT levels, see Tab. S1). This suggests that: (i) chronic antidepressant treatments do not block stress-induced rise of CORT, as found previously [21,22, but see also ref. 23); (ii) the site of action of antidepressants in the modulation of stress-induced glutamate release is downstream of CORT release. In order to assess whether in P/FC the increase of glutamate release is dependent on the binding of CORT to receptors, we treated the rats with RU 486, a selective antagonist of GR, or RU 28318, a selective antagonist of MR, both administered 30 min before the stress session (50 mg/kg s.c.; Fig. 3C–D). RU 486 completely prevented the increase of glutamate release induced by FS-stress, with a significant effect of RU 486 (F_1,12_ = 10,81; p<0.01) and a significant interaction between RU 486 and stress (F_1,12_ = 19,37; p<0.001) (Fig 3C). No significant effects were found for RU 28318 and RU 28318/stress interaction, with only significant effect of stress (F_1,12_ = 20.91; p<0.001) (Fig. 3D). These results suggested that in P/FC FS-stress dependent up-regulation of glutamate release is mediated by increased CORT release and increased stimulation of membrane GR.

### The increase of glutamate release induced in P/FC by footshock stress is accompanied by accumulation of SNARE complexes in the presynaptic membrane

Similar to other cortical areas, glutamatergic neurons and synapses are predominant in P/FC, amounting to up to 80% of total synapses [16]. Moreover, we have shown here that FS-stress significantly increased glutamate release but did not change the release of GABA in P/FC. For these reasons molecular findings observed in presynaptic machinery can be correlated to functional findings (e.g., glutamate release) as done previously [13]. First, we measured the general expression of the SNAREs (synaptobrevin, syntaxin-1 and SNAP-25, the three proteins forming the core exocytotic SNARE complex) as well as their levels in synaptosomes; no changes were found in all rat groups (not shown). Second, we investigated the effect of drug treatments (without stress) on presynaptic SNARE complexes. We have shown previously that in HPC antidepressant-induced reduction of glutamate release is accounted for by changes in protein-protein interactions regulating the assembly of the SNARE complex [13], and by reduction of complex accumulation in presynaptic membranes [16]. However, we found here that the drug treatments did not modify the key interaction syntaxin-1/Munc-18 in P/FC (Fig. S1, see Methods S1). The SNARE complex is SDS-resistant and can be visualized by loading in SDS-PAGE samples of purified presynaptic membranes treated at 25°C instead of 100°C, performing Western blot with antibody for syntaxin-1, and quantitating the complex by digital densitometry [24]–[26]. Two major syntaxin-1-containing complexes, migrating at ∼100 kDa and ∼80 kDa, were detected (Fig. S2A). First, we measured SNARE complexes in drug-treated rats in basal conditions (without stress); we found that in P/FC chronic antidepressant treatments did not induce significant changes in the amount of SNARE complexes (Fig. S2), in line with the lack of changes in depolarization-evoked glutamate release (see Fig. 2A–D).

**Figure 4:**
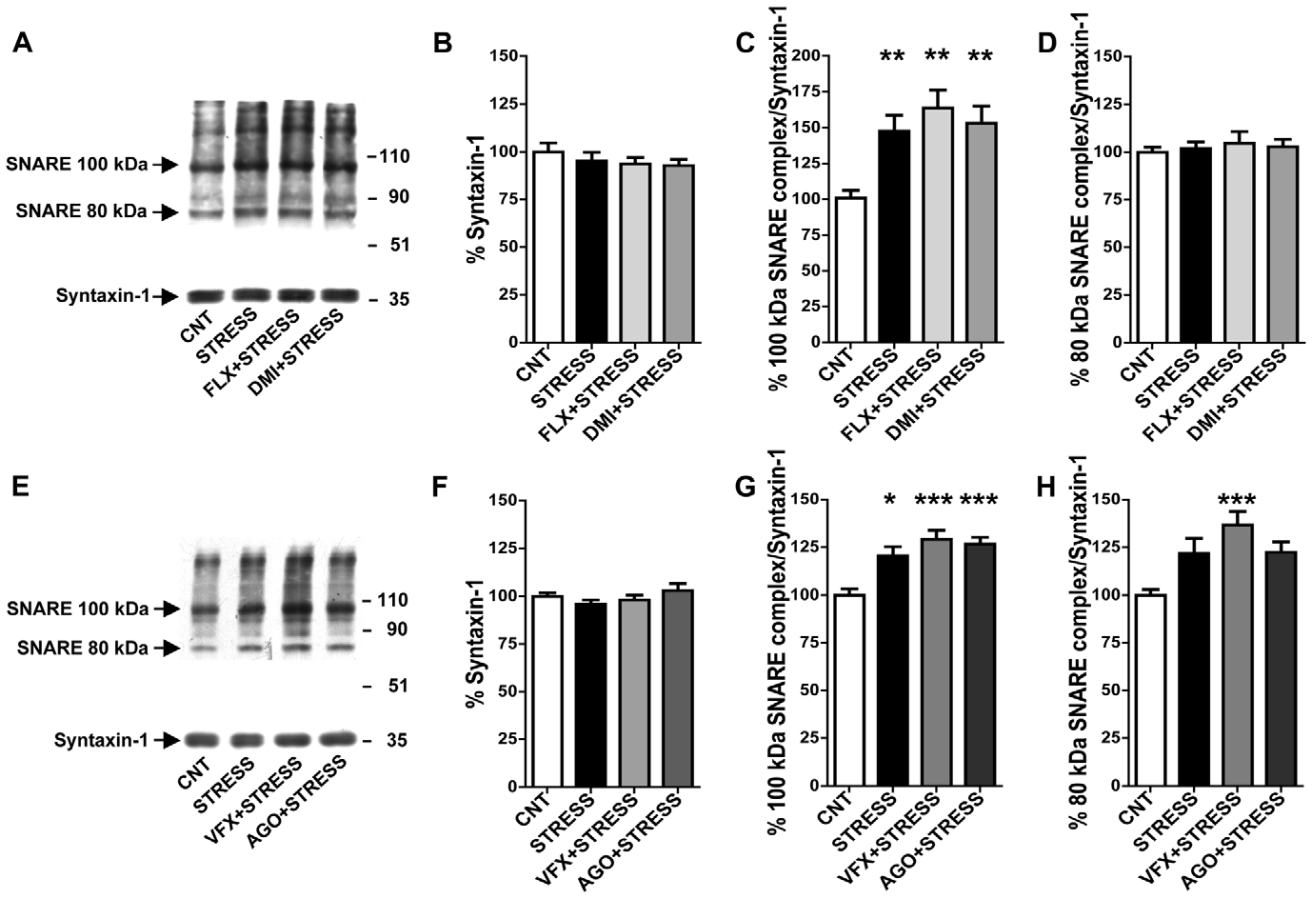
The increase of glutamate release induced in P/FC by acute footshock stress is accounted for by accumulation of SNARE complexes in presynaptic membranes of prefrontal/frontal cortex. A) Representative SNARE complexes of P/FC synaptosomes of vehicle-treated (CNT), subjected to FS-stress (STRESS) and chronically treated with FLX or DMI and then subjected to FS-stress (FLX+STRESS and DMI+STRESS) rats. B) Quantitation of syntaxin-1 in the rat groups as in (B). Data are expressed as means±SEM. C) Quantitation of normalized 100 kDa SNARE complex. Data expressed as above. Each single SNARE complex was normalized on monomeric syntaxin-1 in the same lane. **p<0.01 vs CNT, Newman-Keuls post-hoc tests following one-way ANOVA (n = 6–8 rats/group). D) Quantitation of normalized 80 kDa SNARE complex. E) Representative SNARE complexes of P/FC synaptosomes of vehicle-treated (CNT), subjected to FS-stress (STRESS) and chronically treated with venlafaxine (VFX) or agomelatine (AGO) and then subjected to FS-stress (VFX+STRESS and AGO+STRESS) rats. F) Quantitation of syntaxin-1 in the rat groups as in (F). Data expressed as above. G) Quantitation of normalized 100 kDa SNARE complex. *p<0.05 vs CNT, ***p<0.001 vs CNT; Statistics as above (n = 8–12 rats/group). H) Quantitation of normalized 80 kDa SNARE complex. ***p<0.001 vs CNT; Statistics as above.

Instead, FS-stress induced rapid and significant changes in the amount of SNARE complexes, in line with the rapid increase of glutamate release (Fig. 4). The stress induced accumulation of 100 kDa complexes in presynaptic membranes of P/FC, whether the rats were pretreated or not with antidepressants (Fig. 4A,C,E,G) (F_3,26_ = 6.286; p<0.01 in study 1 and F_3,50_ = 10.09; p<0.0001 in study 2). As observed above for raised CORT levels, these results would suggest that: (i) chronic antidepressant treatments do not block the stress-induced accumulation of SNARE complexes; (ii) the site of action of antidepressants in the modulation of stress-induced glutamate release in P/FC is downstream of SNARE complex formation.

### Footshock stress increases glutamatergic transmission with a dual mechanism and chronic desipramine treatment normalizes it by decreasing release probability

**Figure 5:**
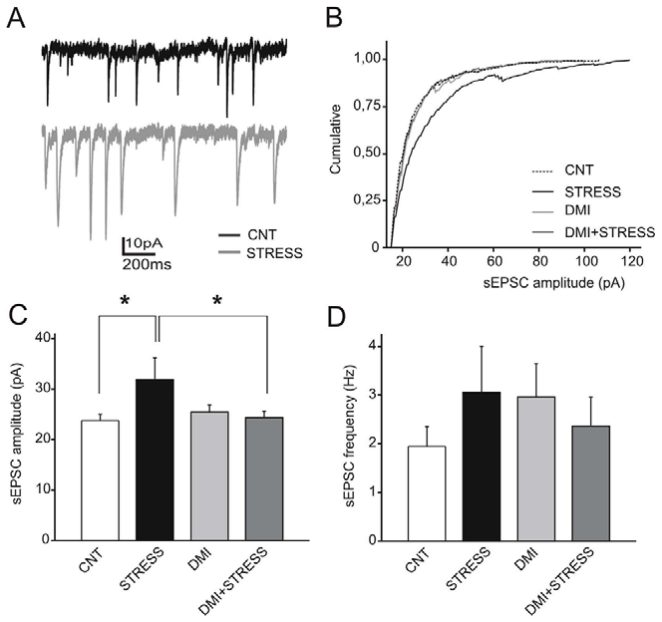
Acute footshock stress enhances the amplitude of spontaneous EPSCs, and desipramine pretreatment completely counteracts this effect. A) Representative traces of EPSCs evoked by spontaneous action potentials (sEPSCs) recorded in pyramidal neurons of layer III of prefrontal cortex obtained from vehicle-treated (CNT) (black trace) and subjected to FS-stress (Stress) (grey trace) rats. B) Mean cumulative analysis of sEPSCs amplitude evaluated in vehicle-treated (CNT) (n = 11), subjected to FS-stress (Stress) (n = 7), chronically treated with DMI (DMI) (n = 9) or chronically treated with DMI and then subjected to FS-stress (DMI+Stress) (n = 9) rats. Cumulative curves were analyzed by using the Kolgomorov-Smirnov test; *p<0.0001 FS-stressed rats vs all the other groups). C,D) Amplitude (C), and frequency (D) of sEPSCs (means±SEM) recorded in neurons obtained from vehicle-treated (CNT) (n = 10), subjected to FS-stress (Stress) (n = 7), chronically treated with DMI (DMI) (n = 8) and chronically treated with DMI and then subjected to FS-stress (DMI+Stress) (n = 9) rats. *p<0.05 Bonferroni post-hoc tests following two-way ANOVA.

The results of the experiments above showed that acute stress rapidly increases the release of glutamate from freshly isolated P/FC synaptic terminals. However, in order to understand whether the same applies to whole tissue, where in vivo circuitry is preserved, we carried out patch-clamp recordings from slices obtained from rats subjected to the same FS-stress paradigm. Spontaneous activity was recorded in pyramidal neurons of layer III of the rat PFC during 15 min periods and EPSCs evoked by spontaneous action potentials (sEPSCs) were selected as currents >15 pA (Fig. 5A). Pyramidal neurons from acutely stressed rats displayed a significant increase in sEPSC amplitude with respect to control neurons (Fig. 5B,C), in the absence of significant effects on sEPSC frequency (D = 0.14; p>0.1; Kolgomorov-Smirnov test; Fig. 5D). Such effect was virtually abolished by previous chronic treatment with the antidepressant DMI (F_3,33_ = 4.474 for the stress- DMI interaction, p<0.05), which was totally ineffective in non-stressed rats (Fig. 5B,C). Cumulative analysis of sEPSC amplitude (Fig. 5B) confirmed that the amplitude distribution of sEPSCs in pyramidal neurons from stressed rats was significantly different from all the other experimental groups (D = 0.23, p<0.0001 and D = 0.21, p<0.0001 vs control and DMI+stress groups, respectively; Kolgomorov-Smirnov test).

**Figure 6:**
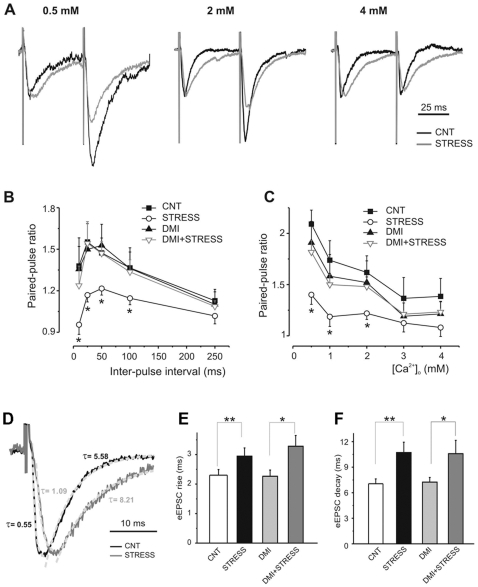
The synaptic facilitation and its Ca^2+^-dependence are significantly reduced in footshock stressed rats and the effect is efficiently prevented by desipramine pre-treatment. A) Specimen paired-pulse eEPSCs at increasing Ca^2+^ concentrations were obtained in vehicle-treated (CNT) and subjected to FS-stress (Stress) rats by stimulating layer V at a time interval of 50 ms and recording from pyramidal neurons. B) The mean paired-pulse ratio (PPR) of the second to the first eEPSC in neurons from vehicle-treated (CNT) (closed squares; n = 11), subjected to FS-stress (Stress) (open circles; n = 11), chronically treated with DMI (DMI) (closed triangles; n = 9) and chronically treated with DMI and then subjected to FS-stress (DMI+Stress) (open triangles; n = 10) rats were plotted as a function of the inter-stimulus interval (from 10 to 250 ms). PPR, at all tested intervals, were significantly lower in cells from FS-stressed rats than in neurons from all the other groups (*p<0.05, Bonferroni post-hoc tests following two-way ANOVA). C) PPR calculated at 50 ms inter-stimulus interval clearly decreased with increasing Ca^2+^ concentrations in neurons of vehicle-treated (CNT) (n = 6), chronically treated with DMI (DMI) (n = 8) and chronically treated with DMI and then subjected to FS-stress (DMI+Stress) (n = 9) rats. On the contrary, neurons from FS-stress rats (n = 6) showed a lower sensitivity to the changes in external Ca^2+^ concentration (*p<0.05, FS-stressed vs all the other groups, Bonferroni post-hoc tests following two-way ANOVA). D) Representative eEPSCs traces recorded in vehicle-treated (CNT) (black trace) and subjected to FS-stress (Stress) (grey trace) rats, showing the rise and decay kinetics fitted by mono-exponential curves. E,F) Rise (E), and decay (F) times (means±SEM) of eEPSCs estimated in neurons obtained from vehicle-treated (CNT) (n = 11), subjected to FS-stress (Stress) (n = 11), chronically treated with DMI (DMI) (n = 8) and chronically treated with DMI and then subjected to FS-stress (DMI+Stress) (n = 8) rats. *p<0.05; **p<0.01, Bonferroni post-hoc tests following two-way ANOVA.

In order to explain the stress-induced increase in sEPSC amplitude, which could be contributed by an increase in the number of the release sites and/or in release probability, PPF was assessed by recording EPSCs evoked by pairs of stimuli applied at increasing interpulse intervals (Fig. 6A). In control neurons, the analysis of the paired-pulse ratio (PPR) of the second to the first eEPSC revealed a clear synaptic facilitation which peaked at short interpulse intervals (Fig. 6B). In contrast to control neurons, pyramidal neurons from acutely stressed rats displayed markedly decreased PPRs, suggesting an increase in release probability.

Interestingly, such effect of acute stress was completely abolished by chronic treatment of the rats with the antidepressant DMI, which was virtually ineffective in the absence of stress. The results indicate that acute stress strongly enhances the neurotransmitter release probability, an effect that is fully normalized by antidepressant pretreatment. To obtain further support to this hypothesis, we estimated the sensitivity of PPR to increasing concentrations of external Ca^2+^ (Fig. 6A,C). In line with the above-mentioned results, neurons from stressed rats showed a markedly lower sensitivity to the changes in the external Ca^2+^ concentration than neurons from control rats, suggesting that the release probability of stressed neurons already reached saturation at physiological Ca^2+^ concentrations. Moreover, the pretreatment with DMI, ineffective in the absence of stress, completely restored the Ca^2+^ sensitivity of the PPR in neurons from stressed rats to values similar to control rats.

Inspection of the shape of EPSCs also revealed a clear slowdown of the kinetics in pyramidal neurons from stressed rats (see, e.g. Figs 5A and 6A). A kinetic analysis of the rise and decay times of eEPSCs revealed that stress significantly increased both the rise (Fig. 6D,E) and the decay (Fig. 6D,F) times of eEPSCs. However, in contrast with the effects on PPR and eEPSC amplitude, chronic pretreatment with DMI was unable to counteract the effect of stress on both parameters (F_3,38_ = 0.001, p = 0.98 and F_3,37_ = 0.177, p = 0.73 for the stress-DMI interaction in the rise and decay times, respectively). Such stress-induced changes in EPSC kinetics strongly suggest a postsynaptic change in glutamate receptors which was not targeted by DMI.

## Discussion

### Acute footshock stress up-regulates glutamate release in prefrontal/frontal cortex by rapid elevation of corticosterone and activation of glucocorticoid receptor

A main finding of the present work was an independent evidence that acute behavioral stress up-regulates stimulation-dependent release of glutamate in P/FC. Although similar data have been previously obtained by a number of microdialysis studies in different areas [7]–[10], independent evidence is scanty. The present results clearly showed with different tecniques that depolarization-dependent release of glutamate is selectively up-regulated by acute stress with respect to GABA release.

A number of previous studies have shown that the effects of acute stress on glutamate release are mediated by raised levels of CORT [20], [27], [28]. Moreover, recent work has shown that acute stress may rapidly increase the level of circulating CORT that, by binding to membrane-located MR and rapid non-transcriptional action, induces the release of glutamate in HPC [11], [12], [29]. Several lines of evidence support the existence of membrane-located G protein-coupled receptors for glucocorticoids in brain, as shown for estrogen receptors [30]. In the present work we obtained evidence that acute stress induces a rapid rise of CORT levels, and that the potentiating effect of CORT on glutamate release in P/FC is mainly mediated by a GR, similar to recent data obtained in PFC with different stressors [31]. However, a participation of MR receptor to this effect of acute stress cannot be completely excluded; similarly, due to the time required to prepare and superfuse the synaptosomes, it is possible that transcriptional effects are also involved here. Interestingly, we found that previous chronic treatment with different antidepressants did not prevent the stress-induced CORT rise in serum, as shown previously [21], [22].

### Mechanisms for the stress-induced up-regulation of glutamate release and transmission

The presynaptic SNARE complex, consisting of a parallel four-helix bundle formed by the SNARE motifs of the three neuronal SNAREs, represents the core of machinery regulating transmitter release [32]–[34]. Previous evidence has shown that the number of SNARE complexes can be increased by chemical treatment of synaptosomes, suggesting a correlation between the accumulation of complexes and the number of vesicles available for release [25]. Moreover, kindling, a model of epileptogenesis associated with sustained enhanced release of glutamate, induces long-term accumulation of SNARE complexes in rat HPC synaptosomes [26]. The present results are in line with this previous evidence, and suggest a mechanism whereby acute stress up-regulates release, possibly by increasing the RRP of glutamatergic vesicles. Interestingly, SNARE complexes were found to be increased in frontal cortex of suicide schizophrenic and depressed individuals [35]. If these data in humans will be confirmed, they will strengthen the idea that excitatory transmission is altered in some psychiatric diseases, and suggest a correlation between cellular/molecular effects of stress and related findings in pathology [3], [36]–[40].

Patch-clamp recordings of pyramidal neurons in the PFC revealed that FS-stress induces a significant increase of the sEPSC amplitude associated with a strong decrease in synaptic facilitation. Such results implicate an increase in release probability in the stress enhancement of the sEPSCs amplitude. This hypothesis is further confirmed by the flat Ca^2+^-dependence of PPF in stressed rats, which indicates that the release probability is already saturated at physiological Ca^2+^ concentrations. On the other hand, the parallel slowdown of both the rise and the decay phases of eEPSCs suggest that a postsynaptic mechanism is also involved in the stress-induced enhancement of glutamatergic transmission. Thus, the data indicate that the effects of stress are two-sided: stress increases glutamate release by increasing the number of SNARE complexes and the probability of release, and also affects the kinetic properties of postsynaptic glutamate receptors. This dual mechanism of action, by increasing the amount of released neurotransmitter and extending the duration of postsynaptic currents, produces a net increase in the depolarizing charge transfer.

### The dampening action of antidepressants on stress-induced glutamate release

Because previous evidence showed that chronic antidepressants reduce glutamate release in basal conditions [13]–[15], we asked here whether these drugs may also dampen the increased glutamate release induced by acute stress. For the first time, we found here that four different antidepressants completely abolished the stress-induced up-regulation of glutamate release, suggesting that this may be a relevant component of the therapeutic action of drugs. Indeed, a dampening of glutamate release could improve the signal to noise ratio, when it becomes compromised by excessive neuronal activation and release due to the action of stress. With regard to the mechanism of this drug effect, as previously discussed [41], we suggest that this is linked to the lack of desensitization of heteroreceptors for 5-HT and NA on postsynaptic cells [42], such as glutamatergic neurons, in the face of the increase in 5-HT- and NA-transmission induced by the chronic treatment with monoamine reuptake inhibitors. For agomelatine, because of its unique receptor profile, the effect on glutamate release is possibly mediated by different mechanisms related to either the melatonin receptor agonism [43] or the 5HT2C receptors antagonism [44], or by a possible synergy between the two properties.

We found that all drug treatments employed here did not block either elevation of circulating levels of CORT or accumulation of SNARE complexes in presynaptic membranes, suggesting that the dampening action of drugs on glutamate release must be on pathways located downstream of these processes and/or on alternative pathways.

Indeed, patch-clamp recordings revealed that DMI pretreatment completely normalized the increase of the sEPSC amplitude and the strong decrease in synaptic facilitation induced by FS-stress, suggesting that the antidepressant counteracts the effect of stress on glutamate release by decreasing the probability of release. Interestingly, DMI was completely ineffective in preventing the effects of stress on the kinetics of eEPSCs, likely occurring at postsynaptic level. Thus, it is possible that DMI leads to a partial prevention of the stress-induced effects, limiting its action at the presynaptic level. We speculate that this action is mediated by changes in the Ca^2+^-triggered process that leads from primed SNARE complexes to membrane fusion. It will be interesting to analyze the interaction of different regulatory proteins with the SNARE complex (e.g., synaptotagmin, complexin, α/β-SNAP, NSF) and investigate whether any of these regulatory steps is affected by the drug treatments.

### Summary

In summary, we found that acute FS-stress induced a rapid up-regulation of depolarization-evoked glutamate release in P/FC, by increasing circulating CORT level, stimulation of GR by CORT and rapid accumulation of presynaptic SNARE complexes in synaptic membranes. Previous chronic treatment with different antidepressants prevented the up-regulation of glutamate release in P/FC, by a mechanism that must be downstream of SNARE complex accumulation. Because antidepressant treatments modify the response to acute stress (measured as glutamate release), we suggest that this mechanism may be related to the therapeutic action of these drugs, particularly when they are prescribed for the therapy of anxiety disorders. It must be mentioned that, although benzodiazepines are increasingly being substituted by antidepressants for the therapy of anxiety, a known mechanism for this action is still lacking.

Because recent developments have identified different sites of regulation of glutamate transmission as potential targets of pharmacological intervention in psychiatric disorders [16], [37], [40], [45]–[48], the study of the action of different psychotropics on the presynaptic protein machinery could uncover additional targets for future therapeutic approaches.

## Materials and Methods

### Footshock stress procedure and drug treatments

All experimental procedures involving animals were performed in accordance with the European Community Council Directive 86/609/EEC and were approved by Italian legislation on animal experimentation (Decreto Ministeriale 124/2003-A). Sprague-Dawley rats (170–200 gr) were used. The footshock (FS)-stress protocol was performed essentially as previously reported [49], (40-min FS-stress; 0.8 mA, 20 min total of actual shock with random intershock length between 2–8 sec). The FS-stress box was connected to a scrambler controller (LE 100-26, Panlab) that delivers intermittent shocks to the metallic floor. Sham-stressed rats (controls) were kept in the stress apparatus without delivering of shocks. In some experiments, the rats were treated 30 min before stress with RU 486, a selective antagonist of glucocorticoid receptor (GR), or RU 28318, a selective antagonist of MR (50 mg/kg s.c.; dissolved in dimethylsulfoxide or propylene glycol, respectively).

The rats were treated chronically (14 days) with antidepressants in two different studies. The drugs were administered for 2 weeks because in previous studies we found that this time allows to measure the drug-induced decrease in depolarization-evoked release in hippocampus of naïve (non stressed rats), and the related molecular changes [13], [50]. Four different drugs were used because each of them represents a different primary mechanism: FLX (selective serotonin reuptake inhibitor), DMI (tricyclic antidepressant), VFX (selective noradrenaline and serotonin reuptake inhibitor), AGO (an altogether different mechanism, agonist for MT1/MT2 melatonin receptors and antagonist for 5-HT2C receptor). Study 1: fluoxetine (FLX; 10 mg/kg), or desipramine (DMI; 10 mg/kg) were delivered in drinking water (vehicle). The average water intake per day for each cage (two rats) was recorded for 4 days before starting and throughout the treatment, and drug solutions were changed every two days according to animals' weight, as reported in previous studies [51], [52]. Rats were subjected to FS-stress 24 h after the last administration and killed thereafter. HPC and the whole frontal lobe, referred to as P/FC were quickly dissected on ice and processed. Study 2: venlafaxine (VFX; 10 mg/kg i.p.), agomelatine (AGO; 40 mg/kg i.p.) or vehicle (hydroxyethylcellulose, 1%, 1 ml/Kg, i.p.) were administered at 5.00 pm (2 h before the start of the dark cycle, 7 pm). Rats were subjected to FS-stress 16 h after the last administration and killed. HPC and P/FC were dissected as above.

Serum was prepared from trunk blood by centrifugation and stored at −80°C. Measurement of serum CORT levels was performed by a commercial kit (Corticosterone EIA kit, Immunodiagnostic System, Boldon, UK).

### Preparation of purified synaptosomes for glutamate/GABA release and synaptic signaling

Purified synaptic terminals (synaptosomes) were prepared by centrifugation on Percoll gradients [53], with minor modifications [13], from fresh brain tissue. Purity of synaptosomes and other subcellular fractions was checked by electron microscopy (not shown) and by measuring subcellular distribution of protein markers, as previously shown [50]. For release experiments, synaptosomes were resuspended in physiological medium with the following composition (mM): NaCl, 125; KCl, 3; MgSO_4_, 1.2; CaCl_2_, 1.2; NaH_2_PO_4_, 1; NaHCO_3_, 22; glucose, 10 (aeration with 95% O_2_ and 5% CO_2_); pH 7.2–7.4. For molecular experiments, synaptosomes were resuspended in lysis buffer: 120 mM NaCl, 20 mM HEPES pH 7.4, 0.1 mM EGTA, 0.1 mM DTT, containing 20 mM NaF, 5 mM Na_2_PO_4_, 1 mM Na_2_VO_4_, and 2 µl/ml of protease inhibitor cocktail (Sigma-Aldrich, Milan, Italy). The synaptic membrane-fraction was prepared as previously reported [50].

### Glutamate and GABA release experiments

Aliquots of synaptosomes (about 100 µg protein) were layered on microporous filters at the bottom of a set of parallel superfusion chambers maintained at 37°C [17], [18]. Superfusion was started at a rate of 0.5 ml/min with standard medium aerated with 95% O_2_ and 5% CO_2_. After 36 min of superfusion, to equilibrate the system, samples were collected according to the following scheme: two 3-min samples (t = 36–39 min and t = 45–48 min; basal outflow) before and after one 6-min sample (t = 39–45 min; stimulus-evoked release). A 90-sec period of stimulation was applied at t = 39 min, after the first sample has been collected. Stimulation of synaptosomes was performed with 15 mM KCl, substituting for equimolar concentration of NaCl. Fractions collected were analysed for endogenous glutamate and GABA content. Amino acid release was expressed as nmol/mg of protein. The stimulus-evoked overflow was estimated by subtracting transmitter content of the two 3-min samples (basal outflow) from release evoked in the 6-min sample collected during and after the depolarization pulse (stimulus-evoked release) [13]. Drug treatment effects were evaluated by comparing the stimulus-evoked overflow in drug-treated animals vs. that calculated in vehicle-treated rats. Appropriate controls were always run in parallel. Endogenous glutamate and GABA were measured by high performance liquid chromatography analysis [13].

### Measurement of SNARE complex in isolated presynaptic membranes

For detection of SDS-resistant SNARE complexes, Western blotting was performed on samples of electrophoresed presynaptic membranes (non-boiled before gel loading) [24]–[26], incubating PVDF membranes containing blotted proteins with monoclonal antibodies for syntaxin-1 1∶5000 (Sigma-Aldrich). The membranes were incubated with anti-mouse secondary antibody 1∶4000 (Sigma-Aldrich), and immunoreactive bands revealed with ECL™ (GE Healthcare, Italy). Membranes were immediately exposed to Hyperfilm ECL™ films (GE Healthcare), and images acquired with the Quantity One software and GelDoc imaging system (Bio-Rad Laboratories, Italy). All bands used were within linear range of standard curves, and normalized for syntaxin-1 monomer levels in the same membrane. Standardization and quantitation of digitalized images were performed with Quantity One software (Bio-Rad).

### Western analysis

Western analysis was carried out as previously described [13], [50], by incubating PVDF membranes, containing electrophoresed and blotted proteins from either synaptic terminals or presynaptic membranes, with monoclonal antibodies for syntaxin-1 1∶8000 (Sigma-Aldrich), synaptobrevin 1∶6000 (Synaptic System, Göttingen, Germany), SNAP-25 1∶8000 (Synaptic System) and β-actin 1∶10000 (Sigma-Aldrich). Following incubation with peroxidase-coupled secondary antibodies, protein bands were detected with ECL™. Standard curves were obtained by loading increasing amounts of samples on gels as previously described [50]. All protein bands used were within linear range, and normalized for β-actin level in the same membrane. Standardization was as above.

### Electrophysiology

Electrophysiological experiments were performed on acute slices of rats subjected to acute stress and/or pretreatment with DMI as described above. When pretreatment with DMI (the only drug tested in these experiments) was performed, brain slices were prepared 24 h after the last administration. Coronal prefrontal cortical slices were obtained from adult male rats as previously described [54]. Whole-cell voltage-clamp recordings from neurons were performed with glass pipettes (∼5 MOhm), pulled from borosilicate glass and filled with the following intracellular solution (mM): 126 Kgluconate, 4 NaCl, 1 MgSO_4_, 0.02 CaCl_2_, 0.1 BAPTA, 15 C_6_H_12_O_6_, 5 HEPES, 3 ATP, 0.1 GTP. Layer III pyramidal neurons in the dorsal part of the medial prefrontal cortex were visualized with a 40× water immersion lens and an infrared camera. Evoked excitatory postsynaptic currents (eEPSCs) were induced in pyramidal neurons of layer III by electrical stimulation of layer V with a bipolar electrode (test pulses at 0.1 Hz, 0.2 ms duration, stimulus intensity fixed at 2/3 of maximal eEPSCs amplitude). When required, solutions at various Ca^2+^ concentrations (0.5–4 mM) were obtained by compensating the NaCl content of the standard recording solution. Voltage clamp data were acquired by using the MultiClamp 700B amplifier (Axon Instruments, Molecular Devices, Sunnyvale, CA, USA) and the pClamp 9.2 software (Axon Instruments).

Rise and decay times of eEPSCs were calculated as the time from baseline-to-peak and from peak-to-30% of the EPSC amplitude, respectively. The amplitude and frequency of sEPSCs were calculated using a peak detector function with a threshold amplitude set at 5 pA and a threshold area at 20 ms*pA. To analyze the paired-pulse ratio (PPR), afferent fibres were stimulated by two brief supraliminar depolarizing pulses applied at 10–250 ms intervals. For each eEPSC couple, PPR was calculated from the equation PPR = I2/I1, where I2 and I1 are the amplitudes of the second and first eEPSC, respectively. Clampfit software was used to analyze eEPSCs.

### Statistical analysis

Statistical analysis was carried out by the unpaired Student's t test when only two experimental groups were involved or by one-way analysis of variance (ANOVA) followed by the Newman-Keuls test for comparisons of data with more than two groups. When the combined effects of stress and treatment were analyzed, two-way ANOVA was used followed by the Bonferroni's post-hoc multiple comparison test. Statistical analysis of the data was carried out using GraphPad Prism4 (GraphPad Software Inc., USA).

Statistical analysis of electrophysiological data was performed with Origin 7.0 (OriginLab Corporation, Northampton, MA) and Sigmastat (Systat Software Inc., Chicago, IL) softwares using one- and two-way ANOVA followed by post-hoc multiple comparison Bonferroni's test. Cumulative curves were analyzed by the Kolmogorov-Smirnov test using the R-CRAN Environment software (http://cran.r-project.org). Figures were edited with Corel Draw 12 (Corel Corporation, Ottawa, Canada). For all analyses a value of p<0.05 was considered statistically significant.

## Supporting Information

Table S1 Absolute corticosterone values (ng/ml) for measurements reported in Fig. 3.
(3.43 MB TIF)

Figure S1 Chronic antidepressant treatment did not modify syntaxin-1/Munc-18 interaction in synaptic membranes of prefrontal/frontal cortex. A) Munc-18 was immunoprecipitated; Munc-18 and syntaxin-1 in the immunoprecipitate were analyzed by Western analysis. Representative immunoreactive bands are shown. CNT, Control; FLX, fluoxetine; DMI, desipramine. Data represent the means±SEM (percentage ratio syntaxin-1/Munc-18) of four separate experiments in duplicate. B) Munc-18 was immunoprecipitated; Munc-18 and syntaxin-1 in the immunoprecipitate were analyzed by Western analysis. Representative immunoreactive bands are shown. CNT, Control; VFX, venlafaxine; AGO, agomelatine. Data represent the means±SEM (percentage ratio syntaxin-1/Munc-18) of four separate experiments in duplicate.
(0.79 MB TIF)

Figure S2 Chronic antidepressant treatment did not modify SNARE complex levels in presynaptic membranes of prefrontal/frontal cortex. A) Representative image of SNARE complexes in synaptosomes visualized by Western blot with a monoclonal antibody for syntaxin-1. The SNARE complex is maintained and visualized only when synaptic membranes are loaded at 25Â°C (25Â° input), while it is disassembled when the samples are heated at 100Â°C before loading (100Â° input). The two SNARE complex forms of approximately 100 and 80 kDa are indicated, as well as the monomeric form of syntaxin-1. B) Representative SNARE complexes in control rats (CNT) and rats chronically treated with fluoxetine (FLX) or desipramine (DMI). C) Quantitation of syntaxin-1 in the rat groups as in (B). Data are expressed as means±SEM. D) Quantitation of normalized 100 kDa SNARE complex. Data expressed as above. Each single SNARE complex was normalized on monomeric syntaxin-1 in the same lane (n = 8 rats/group). E) Quantitation of normalized 80 kDa SNARE complex. F) Representative SNARE complexes in control rats (CNT) and rats chronically treated with venlafaxine (VFX) or agomelatine (AGO). G) Quantitation of syntaxin-1 in the rat groups as in (F). Data expressed as above. H) Quantitation of normalized 100 kDa SNARE complex. Statistics as above (n = 8 rats/group). I) Quantitation of normalized 80 KDa SNARE complex. Statistics as above.
(4.62 MB TIF)

Methods S1 (0.04 MB DOC)
