# Correction: Acute Stress Increases Depolarization-Evoked Glutamate Release in the Rat Prefrontal/Frontal Cortex: The Dampening Action of Antidepressants

**DOI:** 10.1371/annotation/101dd9d3-4e1b-4863-9473-bbfef49c9a1d

**Published:** 2010-01-15

**Authors:** Laura Musazzi, Marco Milanese, Pasqualina Farisello, Simona Zappettini, Daniela Tardito, Valentina S. Barbiero, Tiziana Bonifacino, Alessandra Mallei, Pietro Baldelli, Giorgio Racagni, Maurizio Raiteri, Fabio Benfenati, Giambattista Bonanno, Maurizio Popoli

The affiliations listed for the tenth author, Giorgio Racagni, are incorrect. The correct affiliations are affiliation 1, Department of Pharmacological Sciences, Center of Neuropharmacology and Center of Excellence on Neurodegenerative Diseases, University of Milano, Milano, Italy; and affiliation 4, Istituto di Ricovero e Cura a Carattere Scientifico San Giovanni di Dio - Fatebenefratelli, Brescia, Italy.

