# Supplementary figures and images for: Acute Stress Increases Depolarization-Evoked Glutamate Release in the Rat Prefrontal/Frontal Cortex: The Dampening Action of Antidepressants
Source: PLoS One. 2010 Jan 5;5(1):e8566. doi: 10.1371/journal.pone.0008566 (PMC2797327; doi:10.1371/journal.pone.0008566)

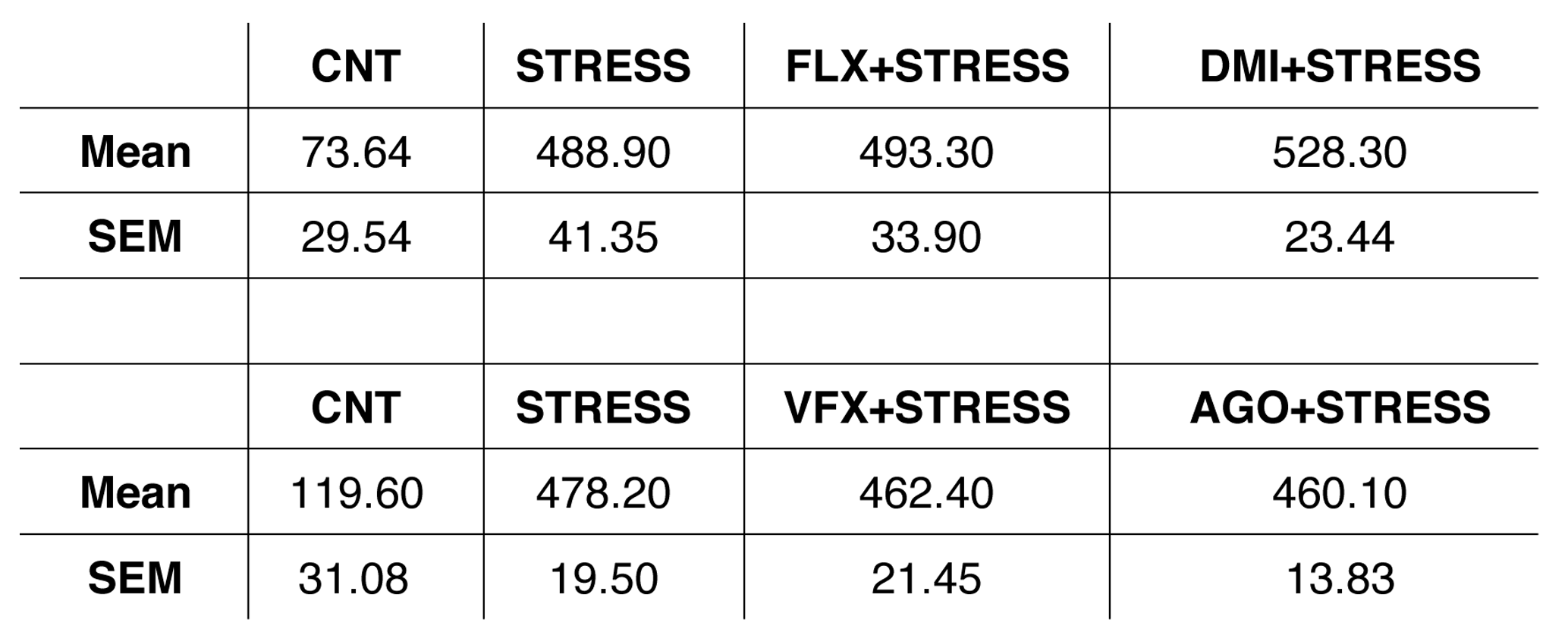

Supplement: Table S1 — Absolute corticosterone values (ng/ml) for measurements reported in Fig. 3. (3.43 MB TIF) [file pone.0008566.s001.tif]

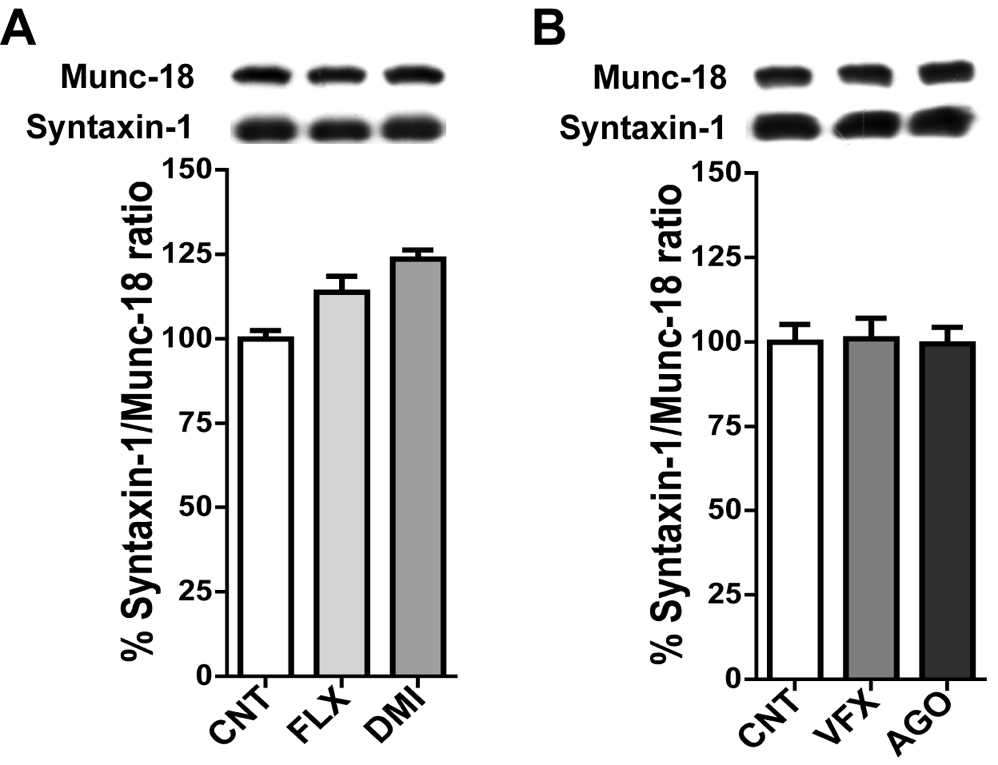

Supplement: Figure S1 — Chronic antidepressant treatment did not modify syntaxin-1/Munc-18 interaction in synaptic membranes of prefrontal/frontal cortex. A) Munc-18 was immunoprecipitated; Munc-18 and syntaxin-1 in the immunoprecipitate were analyzed by Western analysis. Representative immunoreactive bands are shown. CNT, Control; FLX, fluoxetine; DMI, desipramine. Data represent the means±SEM (percentage ratio syntaxin-1/Munc-18) of four separate experiments in duplicate. B) Munc-18 was immunoprecipitated; Munc-18 and syntaxin-1 in the immunoprecipitate were analyzed by Western analysis. Representative immunoreactive bands are shown. CNT, Control; VFX, venlafaxine; AGO, agomelatine. Data represent the means±SEM (percentage ratio syntaxin-1/Munc-18) of four separate experiments in duplicate. (0.79 MB TIF) [file pone.0008566.s002.tif]

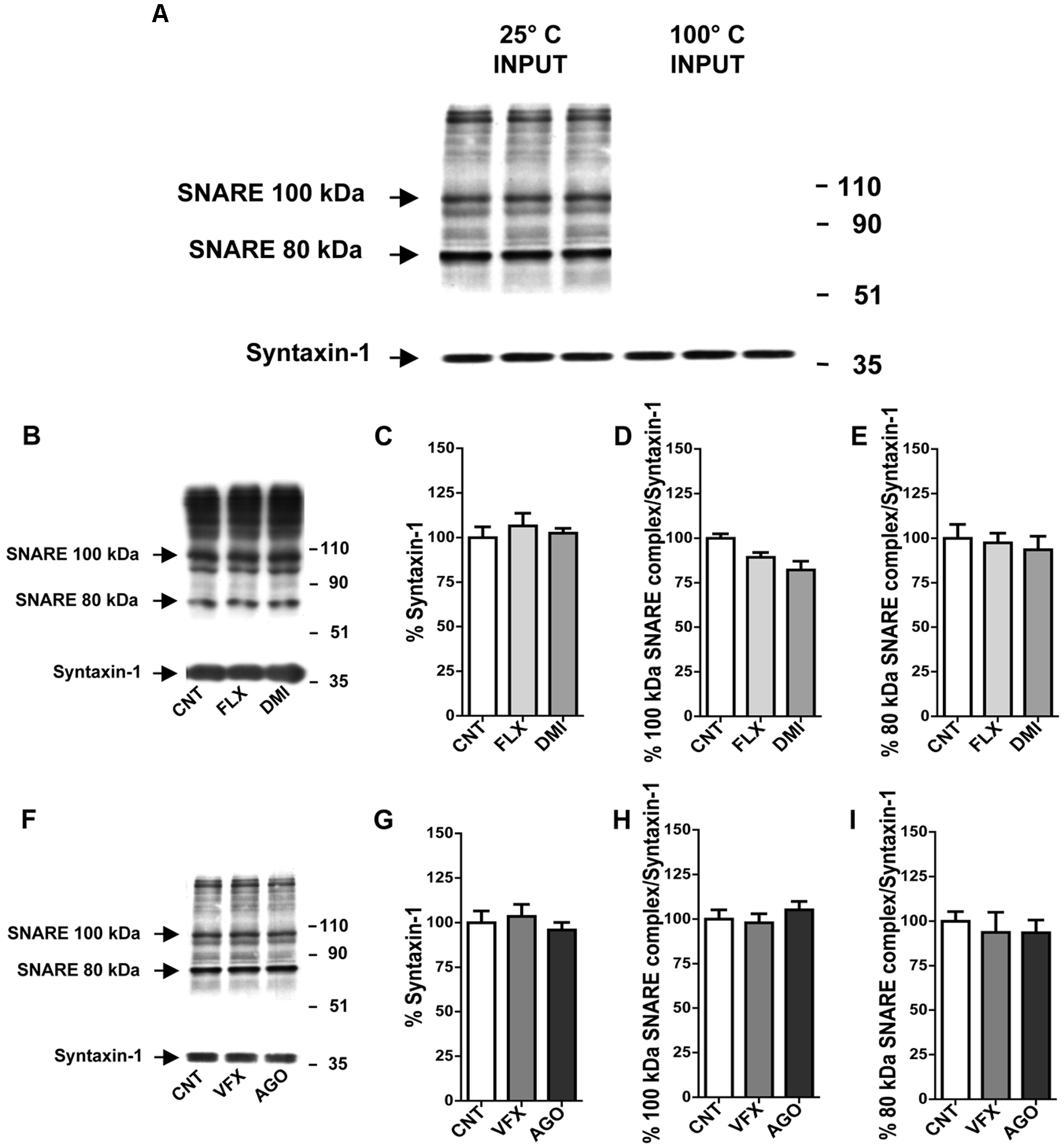

Supplement: Figure S2 — Chronic antidepressant treatment did not modify SNARE complex levels in presynaptic membranes of prefrontal/frontal cortex. A) Representative image of SNARE complexes in synaptosomes visualized by Western blot with a monoclonal antibody for syntaxin-1. The SNARE complex is maintained and visualized only when synaptic membranes are loaded at 25Â°C (25Â° input), while it is disassembled when the samples are heated at 100Â°C before loading (100Â° input). The two SNARE complex forms of approximately 100 and 80 kDa are indicated, as well as the monomeric form of syntaxin-1. B) Representative SNARE complexes in control rats (CNT) and rats chronically treated with fluoxetine (FLX) or desipramine (DMI). C) Quantitation of syntaxin-1 in the rat groups as in (B). Data are expressed as means±SEM. D) Quantitation of normalized 100 kDa SNARE complex. Data expressed as above. Each single SNARE complex was normalized on monomeric syntaxin-1 in the same lane (n = 8 rats/group). E) Quantitation of normalized 80 kDa SNARE complex. F) Representative SNARE complexes in control rats (CNT) and rats chronically treated with venlafaxine (VFX) or agomelatine (AGO). G) Quantitation of syntaxin-1 in the rat groups as in (F). Data expressed as above. H) Quantitation of normalized 100 kDa SNARE complex. Statistics as above (n = 8 rats/group). I) Quantitation of normalized 80 KDa SNARE complex. Statistics as above. (4.62 MB TIF) [file pone.0008566.s003.tif]
