# Supplementary material for: Acute Stress Increases Depolarization-Evoked Glutamate Release in the Rat Prefrontal/Frontal Cortex: The Dampening Action of Antidepressants
Source: PLoS One. 2010 Jan 5;5(1):e8566. doi: 10.1371/journal.pone.0008566 (PMC2797327; doi:10.1371/journal.pone.0008566)
Supplement: Methods S1 — (0.04 MB DOC) [file pone.0008566.s004.doc]

**SUPPORTING INFORMATION**

**Supporting Materials and Methods**

*Coimmunoprecipitations of Munc-18/syntaxin-1*

Aliquots of 100 µg of synaptic membrane fraction (LP1) were incubated in immunoprecipitation (IP) buffer containing (final concentration): 200 mM NaCl, 10 mM EDTA, 10 mM Na_2_HPO_4_, 0.5% SDS, pH 7.4, in a final volume of 200 µl with protease inhibitor mixture (2 µl/ml) (Sigma-Aldrich, Milan, Italy) and antibody against Munc-18 (Becton Dickinson, Rutherford, NJ) overnight at 4° with slow end-over-end rotation. Protein A-Sepharose (Sigma-Aldrich; 10 mg/tube) washed in the same buffer was added and incubation was continued for 2 h at 4°C. The beads were collected by centrifugation and washed four times with 500 µl of the same buffer. Sample buffer (2x) for SDS-PAGE was added, and the mixture was boiled for 3 min. The beads were pelleted by centrifugation, and 25 µl of supernatant was applied to 10% polyacrylamide gels for SDS-PAGE and electroblotted on PVDF membrane for 2 h at 250 mA. Coimmunoprecipitated proteins were detected with primary antibodies for Munc-18 (Becton Dickinson; 1:3000) and syntaxin-1 (Sigma-Aldrich; 1:1000). The membranes were then incubated with secondary antibodies (1:5000) and protein bands were revealed with ECL™.
